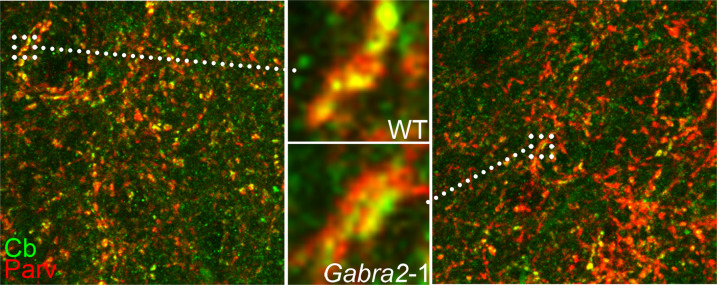# Human *ARHGEF9* intellectual disability syndrome is phenocopied by a mutation that disrupts collybistin binding to the GABA_A_ receptor α2 subunit

**DOI:** 10.1038/s41380-022-01559-x

**Published:** 2022-05-12

**Authors:** Dustin J. Hines, April Contreras, Betsua Garcia, Jeffrey S. Barker, Austin J. Boren, Christelle Moufawad El Achkar, Stephen J. Moss, Rochelle M. Hines

**Affiliations:** 1 Department of Psychology, University of Nevada Las Vegas, Las Vegas, NV USA; 2 Department of Neurology, Boston Children’s Hospital, Boston, MA USA; 3 Department of Neuroscience, Tufts University School of Medicine, Boston, MA USA

Enrichment of collybistin to parvalbumin positive clusters on the soma of pyramidal cells in mouse cortex. Collybistin (green) is strongly enriched at parvalbumin (red) clusters that surround the pyramidal cell soma. Collybistin remains enriched at the parvalbumin clusters on somas in the cortex of *Gabra2*-1 mice suggesting that collybistin is trafficked to these sites independent of interaction with the α2 subunit of GABA_A_ receptors. For more information, please refer to the article by Hines et al. in this issue.